# Remdesivir-Associated Sinus Arrest in COVID-19: A Potential Indication for Close Cardiac Monitoring

**DOI:** 10.7759/cureus.22328

**Published:** 2022-02-17

**Authors:** Grant E Gregory, Hannah M Gregory, Hamza Liaqat, Mina M Ghaly, Kristy D Johnson-Pich

**Affiliations:** 1 Medical School, Alabama College of Osteopathic Medicine, Dothan, USA; 2 Anesthesiology and Critical Care, Alabama College of Osteopathic Medicine, Dothan, USA; 3 Internal Medicine, Wah Medical College, Wah Cantonment, PAK; 4 Internal Medicine, Southeast Health Medical Center, Dothan, USA

**Keywords:** antiviral therapy, drug-induced bradycardia, sinus arrest, remdesivir, covid 19

## Abstract

Remdesivir is an antiviral, nucleoside analog used extensively during the coronavirus-disease 2019 (COVID-19) pandemic with proven efficacy against COVID-19-induced acute respiratory distress syndrome (ARDS). Our case report details the clinical course of a 50-year-old, COVID-19-positive patient who developed sinus arrest after being treated with remdesivir. Within 24 hours of discontinuing remdesivir therapy, the patient’s sinus arrest resolved to a normal sinus rhythm. The findings from our case report add to a growing body of evidence on the cardiotoxic profile of remdesivir. Remdesivir’s ability to cause bradyarrhythmias, and specifically sinus arrest, should be acknowledged when considering the use of this drug in at-risk patients.

## Introduction

Remdesivir is an antiviral agent that has been Remdesivir-Associated Sinus Arrest in COVID-19: A Potential Indication for Close Cardiac Monitoring (FDA)-approved for the treatment of COVID-19 in hospitalized patients [1]. It inhibits SARS-CoV-2 RNA-dependent RNA polymerase (RdRp), which is essential for the replication of a virus [1]. In adult patients who were hospitalized with COVID-19, treatment with remdesivir yielded shorter times to recovery, earlier clinical improvements, and lower mortality rates when compared to treatment with a placebo [2]. However, the employment of remdesivir in the treatment plan of patients does not come without any considerations. Remdesivir has been shown to cause severe bradycardia, which may even be fatal in some patients [1]. Although the mechanism of this cardiotoxic effect is unknown, it has been suggested to be a result of remdesivir’s active metabolite, a nucleotide triphosphate derivative, which may slow sinoatrial node automaticity [3]. Other side effects of remdesivir include hypotension, hepatic transaminase elevations, acute kidney injury, hypersensitivity reactions, nausea, vomiting, and anemia [1,4]. Though it is known that remdesivir can cause bradyarrhythmia in patients, remdesivir-induced sinus arrest is not well-documented in the literature. This case report details the clinical course of a patient who was diagnosed with COVID-19 and developed sinus arrest after being treated with remdesivir.

## Case presentation

**Figure 1:**
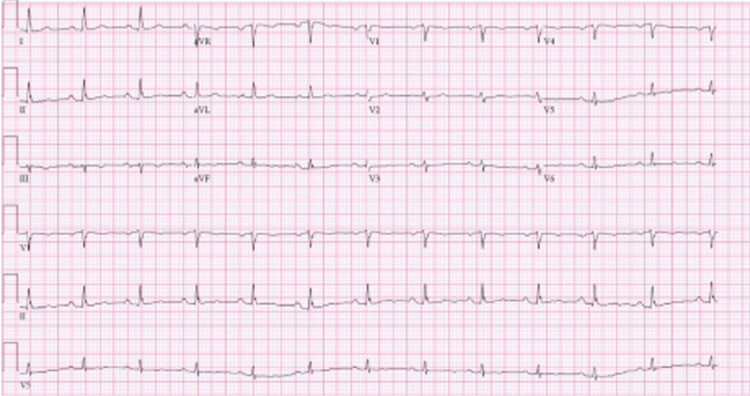
Initial ECG showing normal sinus rhythm.

A 50-year-old female with a known history of coronary artery disease (CAD), hypertension, atrial fibrillation (AF), and chronic obstructive pulmonary disease (COPD) presented to the emergency department (ED) with a two-week history of worsening dyspnea. She also had complaints of mild dry cough and intermittent nausea at the time. She had a known smoking history, but no family history of any arrhythmias was reported. The patient denied any use of rate-controlling medications besides metoprolol for AF. On presentation, the patient was normotensive (blood pressure 137/84 mmHg), had a slightly elevated heart rate (92 beats/min), afebrile (temperature 98.6 degrees Fahrenheit), tachypneic (respiratory rate 24), and hypoxic (O_2_ saturation 78% on room air). The physical exam was unremarkable aside from the lung exam, which revealed bilateral bibasilar crackles. The patient was requiring supplemental oxygen at the time of the initial encounter with 2L via nasal cannula. An electrocardiogram (ECG) at the time of presentation revealed a normal sinus rhythm, as demonstrated in Figure 1.

**Figure 2:**
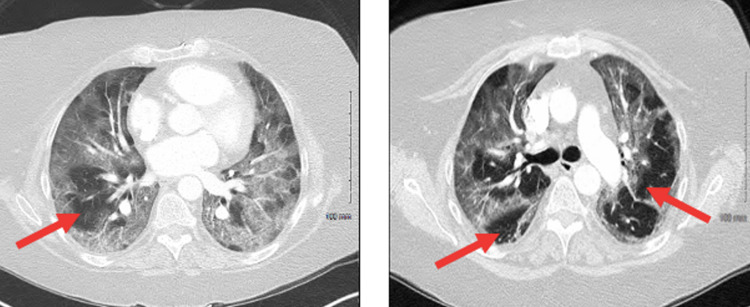
Computed tomography angiography (CTA) images (left, right) of the chest revealing bilateral ground-glass infiltrates, indicative of severe COVID-19 pneumonia.

Lab evaluation revealed normal results for a complete blood count and chemistry panel. However, imaging findings of the chest were consistent with bilateral ground-glass infiltrates without any evidence of pulmonary embolism, as shown in Figure 2. Later on, the patient’s COVID-19 test returned positive. The patient was diagnosed with acute respiratory failure stemming from COVID-19-induced pneumonia as supported by her symptoms, imaging, and positive antigen test.

The patient was started on dexamethasone 6mg daily and was treated empirically with intravenous ceftriaxone 1g daily and azithromycin 250mg for a possible superimposed bacterial infection. The patient also met our hospital criteria for remdesivir therapy. She was initiated on a standard remdesivir dose of 200mg IV on day 1, followed by 100mg IV daily for four days to complete a five-day total course of remdesivir.

**Figure 3:**
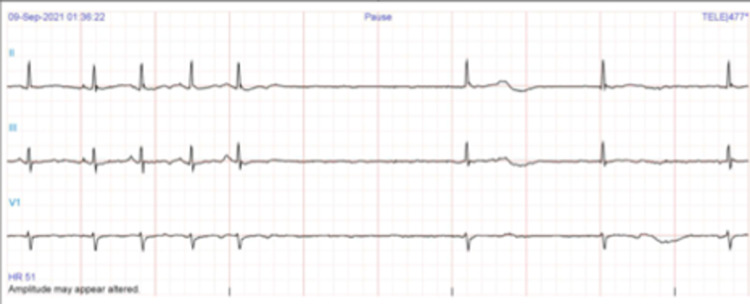
ECG one day after initiation of treatment regimen showing sinus arrest of 3 seconds duration and sinus bradycardia of 30 to 40 bpm.

One day after the initiation of the treatment regimen, the patient was found to have severe sinus bradycardia of 30 to 40 bpm. Metoprolol was identified as a potential culprit and was abruptly discontinued due to the bradyarrhythmia potential of beta-blockers. Despite the discontinuation of the beta-blocker, the patient later developed sinus arrest of three seconds duration, as demonstrated in Figure 3.

**Figure 4:**
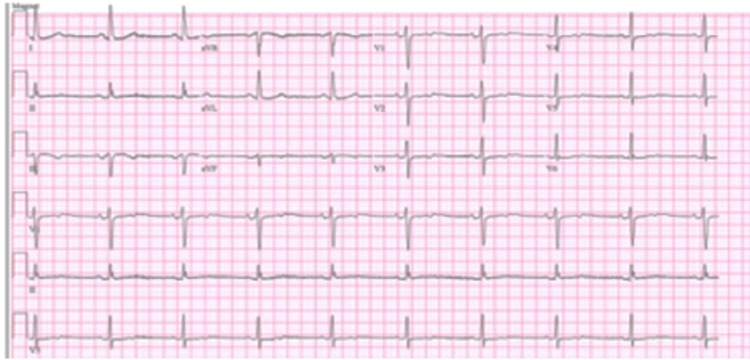
ECG showing resolution of sinus pause and return to the baseline heart rate of 56 bpm 24 hours following remdesivir discontinuation.

After reviewing current literature on the cardiotoxic effects of remdesivir, the decision was made to stop remdesivir. The patient’s bradycardia and sinus arrest ultimately resolved within 24 hours of remdesivir discontinuation, as demonstrated in Figure 4.

## Discussion

Remdesivir is an antiviral agent which works by inhibiting viral RdRp [5]. Originally developed to treat Hepatitis C and other viral outbreaks, remdesivir demonstrated in-vitro activity against coronaviruses during two previous disease outbreaks: MERS-CoV and SARS-CoV [6]. It was labeled as a potential treatment of COVID-19 due to its antiviral effects displaying viral RdRp inhibition [5]. The FDA approved remdesivir for treatment of COVID-19 on October 22, 2020 [7].

Remdesivir is an antiviral agent structurally analogous to adenosine triphosphate [8]. Adenosine is a nucleoside that has antiarrhythmic effects on cardiac conduction cells [8]. Adenosine is most commonly used in the treatment of supraventricular tachycardia due to its intrinsic electrophysiological properties [9]. Adenosine binds to A1 receptors in the heart and acts as a blockade to the atrioventricular (AV) node automaticity [9]. Due to its structural similarity to adenosine, remdesivir has the potential to mimic some of the same antiarrhythmic effects [8,10]. This mechanism suggests plausible causation to remdesivir-associated cardiac conduction abnormalities, as in our patient who developed sinus arrest likely attributed to remdesivir. 

Reportedly, remdesivir has been recently associated with sinus bradycardia. One such study was completed using the World Health Organization Global Individual Case Safety Reports database [11]. The incidence of bradycardia was investigated in COVID-19 patients on remdesivir compared to patients on hydroxychloroquine, lopinavir/ritonavir, tocilizumab, or glucocorticoids. The study found that remdesivir was associated with an increased odds ratio of bradycardic episodes [11]. A different analysis of patients on remdesivir determined that remdesivir was associated with higher rates of bradycardia than the control group [12]. The findings in this study are supported by our case, as our patient developed sinus bradycardia prior to developing sinus arrest while on remdesivir therapy. Another recent study of patients receiving remdesivir underwent daily ECGs to determine arrhythmic changes [13]. Brunetti et al. reported that although patients on remdesivir did show a statistically significant reduction in heart rate, no severe cardiotoxic adverse reactions warranted discontinuation of remdesivir in their patient cohort [13].

A literature review of PubMed and Embase found only one similar case of remdesivir-associated sinus arrest as depicted in the chart below.

**Table 1:** Literature review findings of a similar case of remdesivir-associated sinus arrest.

Authors/Year	Age/Sex	ECG Findings after Initiation of Remdesivir Therapy	Diagnosis	Management	Outcomes	Ref
Sneij et al., 2021	65/Female	Symptomatic sinus bradycardia & sinus pause for 3.5 seconds	Remdesivir-associated bradycardia and sinus pause	Discontinuation of remdesivir	Immediate resolution of sinus pauses and gradual resolution of bradycardia	[10]

In the light of the above discussion on the cardiotoxic profile of this widely used drug during the ongoing COVID-19 pandemic, we suggest close rhythm monitoring of all patients started on remdesivir to avoid any fatal arrhythmias. After carefully ruling out all potential causes of bradyarrhythmias, abrupt discontinuation can be beneficial in such cases. In patients with existing cardiac conduction abnormalities or medications that can induce bradyarrhythmias, the benefits, and risks of remdesivir need to be calculated on an individual basis before initiating treatment. Our case is an excellent contribution to the existing literature on remdesivir adverse effect profiles. Further studies are warranted to learn more about the adverse effects of remdesivir and best practices in management.

## Conclusions

Remdesivir should be acknowledged for its bradyarrythmic potential and should be employed tactically in patients with pre-existing cardiac conditions. Although rare, remdesivir has been shown to cause sinus arrest in some patients. Intensive cardiac rhythm monitoring should be considered for all patients taking remdesivir. In patients who develop sinus arrest or significant bradycardia due to remdesivir, discontinuation of the drug can reduce the risk of potentially fatal arrhythmias and cardiac arrest.
